# Risk factors for treatment failure in late acute periprosthetic joint infection in patients with rheumatoid arthritis treated with surgical debridement – a case-control study

**DOI:** 10.5194/jbji-10-217-2025

**Published:** 2025-07-14

**Authors:** Hendrika M. Schenk, Marine Sebillotte, Jose Lomas, Adrian Taylor, Eva Benavent, Oscar Murillo, Marta Fernandez-Sampedro, Kaisa Huotari, Craig Aboltins, Rihard Trebse, Alex Soriano, Marjan Wouthuyzen-Bakker

**Affiliations:** 1 Department of Internal Medicine, University Medical Center Groningen, Groningen, the Netherlands; 2 Department of Infectious Diseases and Intensive Care Medicine, Rennes University Hospital, Rennes, France; 3 Bone Infection Unit, Nuffield Orthopaedic Centre, Oxford University Hospitals NHS Foundation Trust, Oxford, England; 4 Infectious Disease Service, Bellvitge University Hospital, IDIBELL, Universitat de Barcelona, L'Hospitalet de Llobregat, Barcelona, Spain; 5 Centro de Investigación Biomédica en Red en Enfermedades Infecciosas (CIBERINFEC), Instituto de Salud Carlos III, Madrid, Spain; 6 Infectious Diseases Service, Department of Medicine, Hospital Universitario Marqués de Valdecilla–IDIVAL, Cantabria, Spain; 7 Inflammation Center, Infectious Diseases, Peijas Hospital, Helsinki University Hospital and University of Helsinki, Helsinki, Finland; 8 The Department of Infectious Diseases, Northern Health, Melbourne, Australia; 9 Department of Medicine, Northern Clinical School, The University of Melbourne, Melbourne, Australia; 10 Service for Bone Infections, Valdoltra Orthopaedic Hospital, Ankaran, Slovenia; 11 Faculty of Medicine, University of Ljubljana, Ljubljana, Slovenia; 12 Department of Infectious Diseases, Hospital Clinic of Barcelona, IDIBAPS, Barcelona, Spain; 13 Department of Medical Microbiology and Infection Prevention, University Medical Center Groningen, University of Groningen, Groningen, the Netherlands

## Abstract

**Background**: Patients with rheumatoid arthritis (RA) with late acute periprosthetic joint infections (PJIs) treated with surgical debridement, antibiotics, and implant retention (DAIR) have a high failure rate. We conducted a case-control study to identify risk factors for DAIR failure in this specific patient population. **Methods**: Data from an international multicenter retrospective observational study were used. Late acute PJI was defined as a sudden and acute onset of PJI symptoms occurring more than 3 months after implantation in a previously asymptomatic joint. Cases with RA were matched with cases without RA based on the affected joint. A multivariate Cox regression, stratified for RA, was used to identify risk factors and calculate hazard ratios (HRs) for failure. Subgroup analysis was done to explore the role of immunosuppressive therapy. **Results**: A total of 40 patients with RA and 80 control patients without RA were included. The use or continuation of immunosuppressive drugs was not associated with a higher failure rate. No significant association was found between the duration of symptoms, causative microorganisms, and therapy failure. Bacteremia was an independent predictor for treatment failure (HR of 1.972; 95 % confidence interval, CI, of 1.088–3.573; p = 0.025), and the exchange of modular components was associated with a lower risk of treatment failure (HR of 0.491; 95 % CI of 0.259–0.931; p=0.029 ). **Conclusion**: In patients with RA and a late acute PJI treated with DAIR, bacteremia is an important predictor of treatment failure. Exchanging the modular components seems to be especially important in this patient group and is associated with a lower failure rate.

## 1 Introduction

Periprosthetic joint infection (PJI) is a serious complication causing significant morbidity, mortality, and an increase in health care expenditures after prosthetic joint arthroplasty (Zimmerli et al., 2004). Rheumatoid arthritis (RA) is a chronic systemic connective tissue disease and one of the most common indications of limb joint arthroplasty (Clement et al., 2012). RA has also been shown to be an independent risk factor for the development of a PJI (Hsieh et al., 2013; Kunutsor et al., 2016; Ren et al., 2021). Besides patients having a higher susceptibility to infection due to the disease itself, the use of immunosuppressive medication also contributes to this increased risk (Morrison et al., 2013).

Having RA is also an independent risk factor for treatment failure in acute PJIs treated with debridement, antibiotics, and implant retention (DAIR) (Ghnaimat et al., 2021; Grzelecki et al., 2019; Wouthuyzen-Bakker et al., 2019a). This higher failure rate is particularly evident in late acute PJIs (Wouthuyzen-Bakker et al., 2020). Late acute PJIs are generally a consequence of the hematogenous seeding of bacteria due to infections of the skin, respiratory tract, urinary tract, and other sources as primary focus (Zimmerli et al., 2004).

In a previous analysis of late acute PJIs, we showed that RA patients have a 5 times higher risk of failure compared to non-RA patients (Wouthuyzen-Bakker et al., 2019a). Research focusing on causes of treatment failure in RA patients with a late acute PJI is lacking, yet such work is of clinical value to identify causes of failure and, thus, improve treatment strategies and outcome. Therefore, we conducted a case-control study to identify predictors for the failure of a DAIR procedure in RA patients with a late acute PJI. In addition, we conducted a subanalysis to identify the role of immunosuppressive therapy in treatment failure.

## 2 Material and methods

### 2.1 Study design

Data from a previously described international multicenter retrospective observational study were used (Wouthuyzen-Bakker et al., 2020, 2021). Characteristics of patients with a late acute PJI of the hip or knee between January 2005 and December 2015 were collected from the medical records and anonymized. A minimum number of 10 cases per center were required to participate. Only cases that met the strict definition of late acute PJI were included (Barrett and Atkins, 2014). A late acute PJI case was defined as a patient who developed a sudden onset of symptoms and signs of a PJI, such as acute pain and/or swelling of the prosthetic joint, more than 3 months after the implantation in a previously asymptomatic joint. Patients with a sinus tract and/or symptoms existing for longer than 3 weeks before surgical debridement were excluded. (Parvizi and Gehrke, 2014; Wouthuyzen-Bakker et al., 2019a) A PJI was defined according to the diagnostic criteria described by the Musculoskeletal Infection Society (MSIS) (Parvizi and Gehrke, 2014).

Multiple variables on factors such as patient characteristics, clinical presentation, medical microbiology results, surgical and antibiotic treatment, and outcome were collected and analyzed. In addition to the aforementioned variables, information about the presence of active RA and the use of immunosuppressive therapy was collected.

### 2.2 Clinical outcome

Failure of treatment was defined as follows: (i) the need for prosthesis removal (one- or two-stage exchange, amputation, or a Girdlestone procedure for hips or arthrodesis for knees), (ii) the need for suppressive antibiotic therapy because of persistent clinical or biochemical signs of infection, (iii) relapse of infection with the same microorganism during follow-up, (iv) reinfection with a different microorganism than the initial infection during follow-up, or (v) death due to infection. Death related to PJI was defined as death that occurred during (antibiotic) treatment with no other alternative explanation than an uncontrolled infection. The need for a second debridement during antibiotic therapy was not considered to be a failure, as it was regarded as part of the initial treatment. Patients in whom antibiotic suppressive therapy was prescribed for other reasons than persistent signs of infection were excluded from this study. Follow-up time began at the time of infection diagnosis and ended on the date of failure.

### 2.3 Data analyses

To ensure equal distribution and prevent data loss, cases with RA were matched with controls without RA to create a 1:2 database (Austin, 2010; Rosenbaum and Rubin, 1985). Matching was performed on the affected joint. Characteristics of the cases and controls were summarized using descriptive statistics.

A Cox regression analysis was used to calculate hazard ratios (HRs) for failure, stratified for RA. Stratification allows one to evaluate data from different subgroups with shared characteristics, in this case RA or non-RA. In a univariate analysis, previously selected variables based on clinical and biological relevance (Wouthuyzen-Bakker et al., 2019a) were tested for significance and, when significant, added to a multivariate analysis. A p value of less than 0.05 was considered significant.

### 2.4 Subgroup analysis on immunosuppressive treatment in the RA group

The year of RA diagnosis; the number, type, and dose of immunosuppressive substances; whether the RA was well controlled during infection; whether immunosuppressive medication was discontinued at the time of diagnosis; and when immunosuppressive medication was restarted were additionally collected for the RA cohort. Information on 38 of 40 patients was provided and included in a subanalysis. A binary logistic regression was done to identify whether the number or continuation of immunosuppressive drugs was a risk factor for failure in the RA group. A p value of less than 0.05 was considered significant. Statistical analysis was performed using SPSS, version 26.0 (IBM, Chicago, IL).

## 3 Results

### 3.1 Patient characteristics

In total, 524 patients with late acute PJI were evaluated: 40 had been previously diagnosed with RA, whereas 484 did not have RA. Case-control matching was done to create a 1:2 database, resulting in 40 RA (19 men) and 80 non-RA (41 men) subjects. Matching was done on the affected joint: hip (15 RA and 30 non-RA), knee (21 RA and 42 non-RA), shoulder (3 RA and 6 non-RA), and other (1 RA and 2 non-RA). Table 1 shows the characteristics of both groups. The median age was 71 (interquartile range, IQR, of 60.3–77.5) years for patients with RA and 70 (IQR of 59–79) for patients without RA ( p=0.89 ). Patients with RA used immunosuppressive drugs more often compared to those without RA (27 of 40 vs. 2 of 80; p<0.01 ), and RA patients more often had a serum leucocyte count > $12\times 10^{9}$ L^−1^ at clinical presentation (22 of 38 vs. 26 of 72; p<0.05 ). The time between the implantation of the prosthetic joint and the onset of late acute PJI in RA patients was longer: median 110 (IQR of 41–171) vs. 29 (IQR of 7.5–101.25) months ( p=0.004 ). Causative microorganisms, the number of positive blood cultures, and the duration of antibiotic treatment were comparable between both groups, although the presence of bacteremia approached a significant difference ( p=0.06 ). Failure of treatment occurred in 36 of 80 (45 %) patients in the non-RA group and in 26 of 40 (65 %) patients in the RA group ( p=0.052 ).

**Table 1:** Descriptives for the matched case-control database.

	RA ( n=40 ) (%)	Control ( n=80 ) (%)	p value
Sex	19 men	41 men	0.847
Age in years (median)	71 (IQR of 60.3–77.5)	70 (IQR of 59–79)	0.894
Comorbidity and frailty			
Immunosuppressive drugs	27/40 (67.5)	2/80 (2.5)	<0.01
Clinical presentation			
C-reactive protein > 150 mg L^−1^	22/34 (64.7)	47/74 (63.5)	0.91
Leucocyte > $12\times 10^{9}$ L^−1^	22/38 (57.9)	26/72 (36.1)	0.03
Presence of bacteremia	30/40 (75.0)	46/80 (57.5)	0.06
No. of positive blood cultures	3.0 (IQR of 0–4)	2.0 (IQR of 0–4)	0.446
Polymicrobial infection	5/40 (12.5)	4/80 (5.0)	0.158
Culture-negative	1/40 (2.5)	5/79 (6.3)	0.662
Duration of symptoms in days (median)	5.6 (IQR of 7–14)	4.0 (IQR of 7–14)	0.646
Time between prosthesis	110 (IQR of 41–171)	29 (IQR of 7.5–101.25)	0.004
implantation and diagnosis			
in months (median)			
Causative microorganisms			
Methicillin-sensitive *Staphylococcus aureus*	18/40 (45.0)	28/80 (35.0)	0.323
Methicillin-resistant *Staphylococcus aureus*	4/40 (10.0)	3/80 (3.8)	0.220
Streptococci	10/40 (25.0)	24/80 (30.0)	0.57
Gram-negative	5/40 (12.5)	8/80 (10.0)	0.758
Enterococci	2/40 (5.0)	2/80 (2.5)	0.47
*Candida* spp.	2/40 (5.0)	0/80 (0.0)	0.109
Anaerobe	1/40 (2.5)	1/80 (1.3)	1.00
Antibiotic treatment			
Duration of intravenous treatment in days (median)	22.5 (IQR of 12.5–37.8)	15.5 (IQR of 10–28)	0.119
Duration of oral treatment in days (median)	67.5 (IQR of 27.5–143.3)	58 (IQR of 25.8–90.0)	0.088
Use of rifampicin	18/39 (46.2)	39/79 (49.4)	0.74
Rifampicin for *S. aureus*	17/21 (81.0)	23/30 (76.7)	0.71
Fluoroquinolone for Gram-negative pathogen	3/5 (60.0)	5/8 (62.5)	0.93
Surgical treatment			
Cemented prosthesis	20/29 (69.0)	37/57 (64.9)	0.81
Modular exchange	9/32 (28.1)	29/63 (46.0)	0.092

Values are presented as n / N (%), where n is the number of patients with the characteristic, N is the total number of patients with available data for that variable, and (%) is the corresponding percentage. IQR denotes interquartile range. spp. denotes species.

### 3.2 Case-control analysis, stratified for RA

Table 2 shows the risk factors for failure for both groups when stratified for RA. The presence of bacteremia (HR of 1.870; 95 % CI of 1.093–3.201; p=0.022 ) and modular component exchange (HR of 0.500; 95 % CI of 0.265–0.942; p=0.036 ) were significant in unilateral analysis and added in a bivariate model. In the bivariate analysis, the exchange of modular components was associated with a lower risk of treatment failure (HR of 0.491; 95 % CI of 0.259–0.931; p=0.029 ) in RA patients, whereas the presence of bacteremia was associated with a higher risk of failure (HR of 1.972; 95 % CI of 1.088–3.573; p=0.025 ).

**Table 2:** Risk factors for failure in univariate and multivariate analysis, stratified for RA.

	Univariate				Multivariate		
	HR	95 % CI	p value		HR	95 % CI	p value
Non-RA					1.00		
Duration of symptoms (days)	1.019	0.963–1.078	0.517				
CRP > 150 mg L^−1^	1.202	0.657–2.202	0.551				
Presence of bacteremia	1.870	1.093–3.201	**0.022**		1.972	1.088–3.573	**0.025**
*S. aureus* (MSSA or MRSA)	1.484	0.879–2.503	0.139				
Use of rifampicin	0.942	0.557–1.594	0.824				
Modular component exchange	0.500	0.265–0.942	**0.036**		0.491	0.259–0.931	**0.029**

Note that statistically significant values are shown in bold. Abbreviations used in the table are as follows: RA – rheumatoid arthritis; CRP – C-reactive protein; MSSA – methicillin-sensitive *Staphylococcus aureus*; and MRSA – methicillin-resistant *Staphylococcus aureus*.

### 3.3 Subgroup analyses on immunosuppressive treatment in the RA group

To evaluate whether the number or discontinuation of immunosuppressive drugs in RA patients was associated with treatment failure, we performed subgroup analyses in the RA group. Data on 38 patients (19 men) were included, of which 24 patients had failure of treatment (63 %).

A total of 31 patients (82 %) used at least one type of immunosuppressive drug. A total of 14 patients were on methotrexate (MTX; dose range 7.5–20 mg per week), 19 patients were on (methyl)prednisone (dose range 2–30 mg d^−1^), and 12 patients used disease-modifying antirheumatic drugs (DMARDs; e.g., sulfasalazine, rituximab, hydroxychloroquine, leflunomide, adalimumab, and etanercept), with patients taking a maximum of four different drugs. In 6 patients (17.6 %), all immunosuppressive drugs were stopped during treatment; in 2 cases, immunosuppressive drugs were partly stopped (5.9 %); and in 22 cases, immunosuppressive drugs were not stopped (57.9 %). In one case, data were missing. In 24 cases, the RA was well controlled at time of infection diagnosis; in 10 cases, it was poorly controlled; and information on 4 cases was missing. No significant association was found between treatment failure in RA patients and the number of immunosuppressive drugs (odds ratio, OR, of 1.43; 95 % CI of 0.59–4.35) or the discontinuation of immunosuppressive medication (OR of 0.69; 95 % CI of 0.21–3.90).

## 4 Discussion

We specifically investigated risk factors for failure in patients with RA with late acute PJI treated with DAIR, as the literature on this subject is sparse. In this study, RA patients benefited more from the exchange of modular components. Furthermore, the presence of bacteremia was associated with a higher failure rate in patients with RA compared to patients without RA.

In one of our previous studies, performed in a large cohort of patients with late acute PJI treated with DAIR, the presence of bacteremia during the initial clinical presentation was not associated with a higher failure rate (Wouthuyzen-Bakker et al., 2019). However, the association between bacteremia and treatment failure has been described in a study performed by Kuo et al. (2019). In this cohort of patients with late acute PJI, having positive blood cultures was associated with 4 times higher odds for failure after adjusting for confounding variables (Kuo et al., 2019). The percentage of patients with RA in this study was not described.

In our current study, the presence of bacteremia approached a significant difference between RA and non-RA patients, and its presence was independently associated with treatment failure. This could be due to the high rate of immunosuppressive drugs used in the RA population, which impairs the clearance of bacteremia. Bacteremia indicates the spread of infection and is linked to worse clinical outcomes, especially when caused by highly virulent bacteria that can breach immune defenses, leading to more severe infections.

As a consequence, continuous seeding of bacteria to the prosthetic joint and the subsequent formation of biofilm could complicate the eradication of bacteria on the implant to an even greater extent in immunocompromised hosts compared to controls. These factors may explain our observation that modular component exchange seems to be particularly important in patients with RA. An alternative explanation for why modular component exchange may be of greater importance in the RA population might be that RA patients show a different cellular response to debris caused by prosthetic wear (Vasudevan et al., 2012), which may hamper infection eradication.

We hypothesized that a higher failure rate in RA patients might be caused by a delay in PJI diagnosis due to the suppression of symptoms during immunosuppressive treatment; the mimic of RA flare-ups; and, thus, a delay in clinical presentation (Bari et al., 2013; Dhillon et al., 2009). Moreover, RA increases infection risk due to immune dysfunction, impaired barriers, and delayed responses, allowing pathogens to spread (Listing et al., 2013). Recognition of PJI in patients with an inflammatory joint disease can be challenging, as serological diagnostic markers do not distinguish between active autoimmune disease and bacterial infection (Cipriano et al., 2012; Tahta et al., 2019). Our univariate analysis showed no difference in the duration of symptoms between patient groups, indicating that there was no delay in PJI diagnosis and subsequent surgical intervention. However, our data are not sufficient to substantiate whether the duration of symptoms before clinical presentation between the groups was significantly different.

We also did not find any association between the use of immunosuppressive drugs and treatment failure, although this association between long-term immune suppression and failure in RA was found in the study of Berbari et al. (2006). However, it should be noted that a limited number of cases were included in our study and that all immunosuppressive drugs were combined into one dichotomous variable. The difference in underlying mechanisms, duration of use, or time of administration of immunosuppressive drugs could influence the results (George et al., 2017). Given the prolonged half-life of newer immunomodulating drugs, immune suppression persists after discontinuation. As PJI treatment can last months, further research is needed on the long-term effects of therapy interruption, and an individual approach is crucial, balancing infection risks with RA flare-ups.

In a large proportion of RA patients in our cohort, modular components were not exchanged (70 %). A potential explanation for this could be that the modular components were not available at the time of surgical debridement, which is reflected in the finding that the prostheses of RA patients were older compared to control patients without RA (Table 1). Not exchanging modular parts and, consequently, insufficient surgical debridement was shown to be a factor for late acute failures in this cohort, particularly for RA patients. Therefore, when modular component exchange cannot be performed, extraction of the prosthesis should be considered. Our database does not specify the method used by the surgeon to clean the retained mobile components; thus, the influence of the cleaning process is unknown. However, when extraction is impossible, thorough cleaning of the mobile components may help preserve the prosthesis and prevent treatment failure.

Our findings are supported by several other studies. In a systematic review by Desai et al. (2024), 401 PJI patients with RA treated with DAIR ( n=204 ), two-stage exchange ( n=123 ), and resection arthroplasty ( n=74 ) were analyzed. They demonstrated a significantly lower failure rate for two-stage exchange (26.8 %) compared to one-stage exchange arthroplasty/revision (39.2 %) and DAIR (60.1 %). In the majority of the patients in this study who underwent DAIR, surgical debridement was performed with retention of components, indicating that modular components were not exchanged in this patient population. In another study (Berbari et al., 2006), patients with two-stage exchange had chronic PJI more often compared to those treated with DAIR, making extrapolation of the results to acute PJIs difficult. Nonetheless, overall treatment success for two-stage exchange was much higher compared to surgical debridement.

This study has several strengths and limitations. A major strength is the cooperation between multiple centers, creating the opportunity to study this specific patient group of late acute PJIs with RA. However, the relatively small sample size limits the possibilities with respect to additional important (sub)analyses. Overcoming the problem of the small sample size and the relatively long period of data collection is challenging and maybe even unfeasible, given the nature of the population and the type of disorder studied. In one of the largest studies, if not the largest, on PJI in RA patients (with 200 patients), data were included over a time period of 27 years (Berbari et al., 2006). The patient population is small due to the rarity of the complication, and (compared to early acute PJI) the incidence of late acute PJI is also lower (Weinstein et al., 2023).

Another limitation was the lack of detailed data on RA and its disease activity, due to the retrospective character of the study, and the variety with respect to the use of immunosuppressive drugs in the RA group. Some RA patients did not use immunosuppressive drugs at all, whereas others used up to four different drugs. The type of drugs varied within the group, which could explain why no association was found with failure. A final limitation was the lack of data about the consistency and thoroughness of surgical debridement, which is an important predictor of DAIR success. However, all centers participating in the study are experienced centers in the treatment of PJI.

## 5 Conclusions

The goal of this study was to identify predictors of failure in patients with RA and late acute PJIs treated with DAIR. We conclude that the presence of bacteremia during initial presentation is a significant risk factor. Our results also suggest that modular component exchange is important and associated with a lower risk of treatment failure. Although studies exploring the difference between DAIR with or without modular exchange in the RA population are lacking, prothesis removal seems to be associated with a higher success rate (Desai et al., 2024). Based on the current literature, extraction of the prosthesis should be considered when modular component exchange is not possible. For future studies a multicenter prospective case-control study design could help to understand whether RA patients would benefit more from DAIR with modular component exchange, a one-stage revision, or a two-stage revision. It would be interesting to see if the presence of bacteriemia and other factors, e.g., the age of the prothesis or the use of immunosuppression, influence in the outcome in all different treatments.

## Data Availability

Data can be made available upon request from the corresponding author.

## Team list

Jaime Esteban (Department of Clinical Microbiology, Instituto de Investigación Sanitaria – Fundación Jiménez Díaz University Hospital, Universidad Autónoma de Madrid (IIS-FJD, UAM), Madrid, Spain), Marjan Wouthuyzen-Bakker (Department of Medical Microbiology and Infection Prevention, University Medical Center Groningen, University of Groningen, Groningen, the Netherlands), Efthymia Giannitsioti (Department of Infectious Diseases, Northern Health, Melbourne, Australia), Craig Aboltins (LAIKO General Hospital, First Department of Propaedeutic and Internal Medicine, Medical School – National and Kapodistrian University, Athens, Greece), and Barbara Hasse (Infectious Diseases and Hospital Epidemiology Zurich, Switzerland).
